# Visual Recovery and Neurological Stabilization Following Miglustat Treatment in Pediatric CLN3 Disease

**DOI:** 10.1177/08830738251374538

**Published:** 2025-09-18

**Authors:** Alice E. Dutton, Ineka T. Whiteman, Michael M. Jones, Katie E. Geering, Soheil Afshar, Alexandra M. Johnson, John R. Grigg

**Affiliations:** 1 Department of Ophthalmology, 371501 Sydney Children's Hospital Network, The Children's Hospital at Westmead, Westmead, New South Wales, Australia; 2 Save Sight Institute, Faculty of Medicine and Health, University of Sydney, Sydney, New South Wales, Australia; 3 638051 Batten Disease Support & Research Association (BDSRA) Australia, Shelly Beach New South Wales, Australia; 4 BDSRA Foundation, Columbus OH, USA; 5 512473 Beyond Batten Disease Foundation, Austin TX, USA; 6 Department of Orthoptics, 371501 The Sydney Children's Hospital Network, Westmead, New South Wales, Australia; 7 Department of Psychology, 63623 Sydney Children's Hospital, Randwick, New South Wales, Australia; 8 Department of Neurology, 63623 Sydney Children's Hospital, University of New South Wales, Randwick, New South Wales, Australia; 9 Eye Genetics Research Unit, 58454 Children's Medical Research Institute, Save Sight Institute, The University of Sydney and Sydney Eye Hospital, Sydney, New South Wales, Australia

**Keywords:** CLN3 disease, Juvenile Batten disease, miglustat, ophthalmology, pediatric

## Abstract

Neuronal ceroid lipofuscinosis type 3 (CLN3) disease is a rare, life-limiting pediatric neurodegenerative disorder with no approved disease-modifying therapy. We conducted a prospective case report from October 2023 to April 2025 involving two female siblings with genetically confirmed CLN3 disease (homozygous for the common 1 kb deletion). Both patients were treated with oral, weight-based miglustat for 18 months. Miglustat was supplied as off-label use in the absence of a therapeutic alternative for this severe neurodegenerative disorder. Clinical outcomes were assessed using comprehensive ophthalmologic evaluation, the Unified Batten Disease Rating Scale (UBDRS), and the Vineland Adaptive Behavior Scales, Third Edition (Vineland-3). At the time of report, patients were aged 13 and 10 years. Both had been diagnosed at age 7 years and commenced miglustat at ages 11 and 9 years, respectively. Over the treatment period, both patients demonstrated improvement in visual acuity and clinical stabilization on the Unified Batten Disease Rating Scale. One patient showed measurable improvement in adaptive functioning as assessed by Vineland-3. No significant adverse effects were reported. These preliminary findings suggest potential short-term clinical benefit of miglustat in pediatric patients with CLN3 disease, particularly when initiated early in the disease course. Further studies involving larger cohorts and longer follow-up are warranted to evaluate the safety and long-term efficacy of miglustat in this population.

Neuronal ceroid lipofuscinosis type 3 (CLN3 disease), also known as juvenile Batten disease, is a rare pediatric lysosomal storage disease caused by biallelic pathogenic variations in the *CLN3* gene (OMIM 607042).^1^ Patients are typically diagnosed during their first decade of life, often following an otherwise healthy period of early childhood development. Vision loss is typically the first presenting symptom,^2–7^ with an average age of onset ranging from 5.4 ± 1.4 years (mean ± standard deviation)^2^ to 6.5 ± 1.1 years.^4^ This is followed by a relatively rapid visual decline and complete blindness by around age 10-12 years.^2,3,5^ Cognitive impairment and notable behavioral changes can appear around the same time, at 6-8 years,^8^ followed by motor decline (10-12 years),^2,3^ seizures (10-12 years),^8^ speech and language decline (11-17 years),^8,9^ feeding and swallowing difficulties,^4^ and ultimately premature death, typically in the third decade of life.^3,8^ At present there is no approved disease-modifying treatment, leaving affected individuals and their families with a significant and urgent unmet need.

Miglustat is a substrate reduction therapy approved by Therapeutic Goods Administration (TGA) in Australia^10^ for use in lysosomal storage disorders including Gaucher disease and Niemann-Pick type C disease. It has been investigated preclinically and in a phase I/II clinical trial in 6 patients 17 years or older as a potential treatment for CLN3 disease.^11,12^ We hypothesized that commencement of miglustat treatment early in the disease course may alter the natural history of progressive, irreversible neurologic and retinal degeneration associated with CLN3 disease.

We present a report of 2 cases of pediatric sibling patients with classical CLN3 disease who have been treated off-label on a compassionate use basis with miglustat for 18 months, highlighting its impact on visual and neurologic function. To our knowledge, this is the first report of patient outcomes including visual function in children (under 17 years) with CLN3 disease treated with miglustat.

## Patients and Methods

Two pediatric patients with genetically confirmed CLN3 disease were prospectively followed for 18 months in the Sydney Children's Hospital Network (Children's Hospital Westmead and Sydney Children's Hospital), Sydney, Australia, between October 2023 and April 2025. The project was approved by Sydney Children's Hospitals Network Human Research Ethics Committee. The parents of the children provided written informed consent for publication. Oral miglustat therapy (Zavesca 100-mg tablets, used off-label) was commenced in October 2023 and dosed according to body surface area (rounded to the nearest whole tablet) and titrated over 1 month to maximum 600 mg/d in 3 divided doses (as approved for treatment of Niemann-Pick type C disease).^10^

Main outcome measures have been reported using Unified Batten Disease Rating Scale (UBDRS), Vineland Adaptive Behavior Scales, Third Edition (Vineland-3) Comprehensive Parent/Caregiver Form, and comprehensive ophthalmologic assessments including visual acuity (Sheridan Gardiner Test), ultra-widefield fundus pseudocolor imaging and ultra-widefield–fundus autofluorescence using Optos (Optis plc, Dunfermline, United Kingdom), and optical coherence tomography using Heidelberg Spectralis (Heidelberg Engineering, Germany) and Zeiss Cirrus (Carl Zeiss Meditec, Dublin, CA).

Formal visual function assessment was undertaken prior to the commencement of miglustat and then 3-monthly. Baseline and follow-up neurologic assessments were performed at 0, 6, 11 and 17 months for the Unified Batten Disease Rating Scale, and at 0, 11 and 18 months for Vineland. Tests were performed by the same providers, in a consistent environment and all results reported in medical records.

Visual acuity testing, and hence objective monitoring of disease, can be challenging as the disease progresses as a result of both poor patient cooperation and extremely low visual acuity. Visual acuities were converted to logMAR. If visual acuity was recorded as “hand motion,” it was converted to logMAR using the Freiburg Visual Acuity Test.^13^

Visual electrophysiology was performed at baseline for 1 patient. Testing strategies included pattern electroretinogram and full-field electroretinogram using Espion (Diagnosys, Lowell, MA). Visual electrophysiology was performed according to International Society for Clinical Electrophysiology of Vision (ISCEV) standards, including the shortened pediatric protocol.^14^

## Results

### Patient 1

A 4-year-old White female was referred to an ophthalmologist after failing the Statewide Eyesight Preschooler Screening (StEPS) program.^15^ She had no significant medical or ocular history, and no relevant family history. Her uncorrected visual acuity was right 0.4 and left 0.3 logMAR (20/50 and 20/40). Ocular motility was normal with normal stereopsis. A cycloplegic refraction revealed a spectacle correction of right +2.0/+1.0 × 100 and left +1.50/+1.25 × 120. Anterior- and posterior-segment examinations were normal. Glasses were prescribed. Review 9 months later revealed best corrected visual acuity to be right 0.2 and left 0.3 logMAR (20/30 and 20/40).

**Figure 1.**
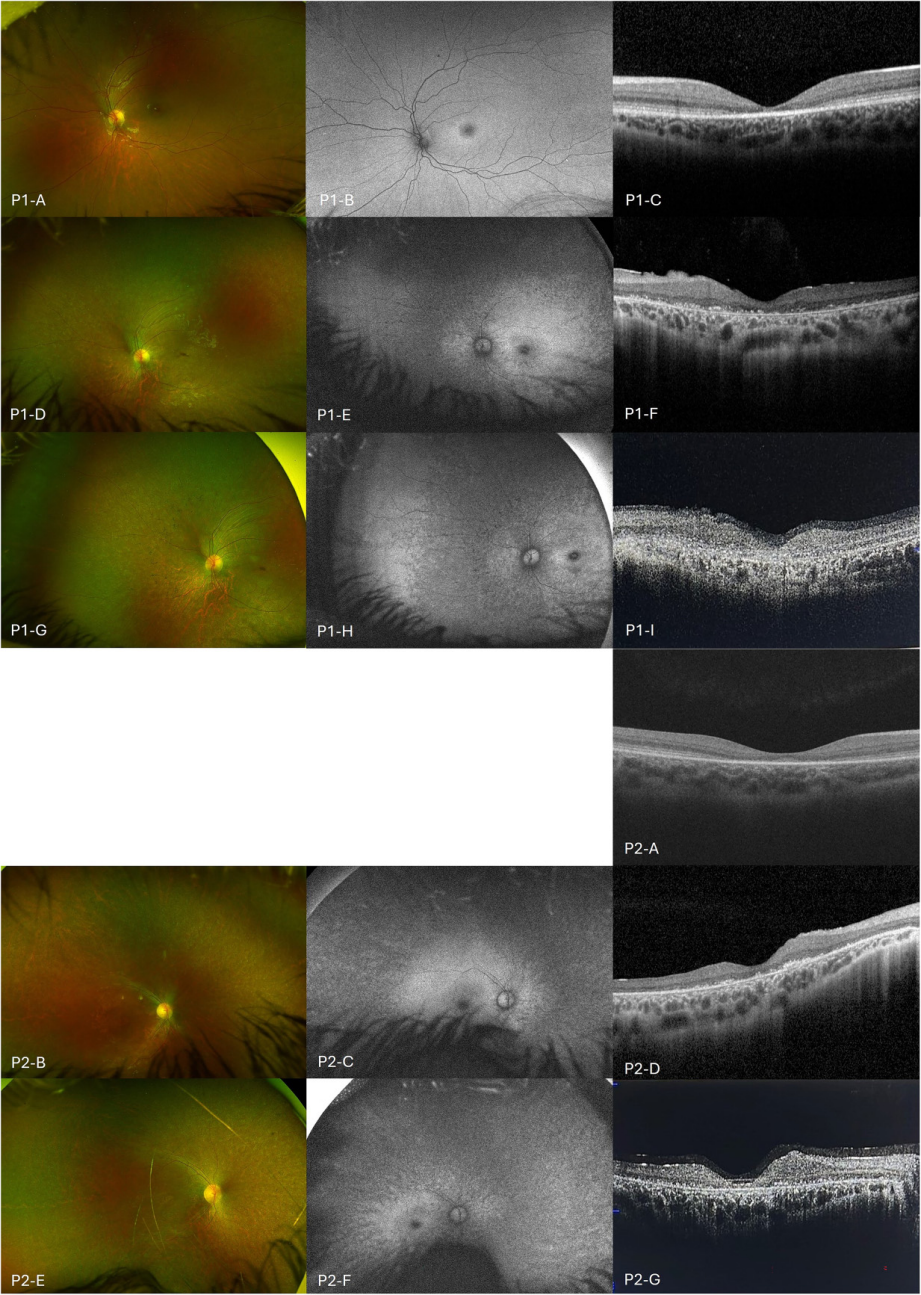
Figure 1. Multimodal Retinal Imaging. Multimodal Retinal Imaging or Patient 1 (P1) and Patient 2 (P2). UWF Fundus Pseudocolor Photograph, UWF-FAF, and Macula OCT. (**P1-A** to **P1-C**) Left Eye, Age 7 Year 1 Month. (**P1-A**) UWF Pseudocolor Image Shows Start of a Bull's-eye-type Appearance with Atrophy at the Macula. (**P1B**) The UWF-FAF Shows an Early Diffuse Ring of Hyper-AF Around the Fovea. (**P1C**) The OCT Shows Loss of the Outer Retinal Structures Including the Ellipsoid Zone at the Macula, but there is Sparing in the Peripheral Macula. (**P1-D** to **P1-F**) Left Eye, Age 11 Years 6 Months (Prior to Commencement of Miglustat). (**P1-D**) UWF Pseudocolor Image Shows Progressed Retinal Atrophy, Extending Beyond the Vascular Arcades to the Periphery. (**P1-E**) UWF-FAF Shows Corresponding Hypo-AF in a Bull's-eye Pattern at the Macula and Extending Beyond the Vascular Arcades Nasally. (**P1-F**) OCT Demonstrates Significant Outer Retinal Disruption Involving the Extent of the Scan. (**P1-G** to **P1-I**) Left Eye, Age 13 Years 2 Months (After 18 Months of Miglustat Therapy). (**P1-G**) UWF Pseudocolor Image Shows Widespread Retinal Atrophy. (**P1-H**) UWF-FAF Shows Increased Mottled Hypo-AF Extending Inwards from the Mid-periphery to the Vascular Arcades. (**P1-I**) OCT Shows Similar Outer Retinal Atrophy with Loss of Inner Retinal Structures at the Fovea; Fixation is Challenging Because of Poor Visual Acuity. (**P2-A**) Right Eye, Age 7 Years 0 Months; the OCT Shows Loss of the Outer Retinal Structures Including the Ellipsoid Zone at the Perifoveal Macula in a Bull's-eye Pattern; Baseline UWF Pseudocolor and UWF-FAF Images were Unable to be Obtained Due to Patient Cooperation. (**P2-B** to **P2-D**) Right Eye, Age 9 Years 1 Month (Prior to Commencement of Miglustat). (**P2-B**) UWF Pseudocolor Image Shows Widespread Retinal Atrophy Extending to the Periphery. (**P2-C**) UWF-FAF Shows Hypo-AF at the Macula and Extending Beyond the Vascular Arcades. (**P2-D**) OCT Shows Progression in Outer Retinal Atrophy with Significant Ellipsoid Zone Disruption. (**P2-E** to **P2-G**) Right Eye, Age 10 Years 9 Months (After 18 Months of Miglustat Therapy). (**P2-E**) UWF Pseudocolor Image Shows Widespread Retinal Atrophy. (**P2-F**) UWF-FAF Shows Increased Hypo-AF, which has Progressed from the Midperiphery to the Vascular Arcades. (**P2-G**) OCT Demonstrates Persistent Outer Retinal Atrophy and Disruption; Fixation is Challenging Because of Poor Visual Acuity. OCT, Optical Coherence Tomography; P1, Patient 1; P2, Patient 2; UWF-FAF, Ultra-widefield-fundus Autofluorescence.

Review at age 7 years 0 months identified a history of subtle visual decline over the preceding 6 months. Best corrected visual acuity was reduced to 0.6 logMAR (20/80) both eyes. Funduscopy revealed a bull's-eye maculopathy. Ultra-widefield fundus pseudocolor imaging and ultra-widefield–fundus autofluorescence documented a bull's-eye maculopathy (Figure 1, P1-A and P1-B). Optical coherence tomography using Zeiss Cirrus (Carl Zeiss Meditec) confirmed ellipsoid zone (EZ) disruption at the fovea (Figure 1 P1-C). Visual electrophysiology was performed. The pattern electroretinogram was very noisy and it was difficult to detect recordings from background noise. A flash electroretinogram was suggestive of an electronegative waveform with cone dysfunction. Targeted gene sequencing was undertaken, which reported homozygous pathogenic variants *CLN3* c.461-280_677+382del966, consistent with a diagnosis of CLN3 disease.

There was a history of clumsiness and motor dyspraxia, as well as behavioral issues, including sudden outbursts of anger, acting out, and biting. The patient was having difficulty with arithmetic at the time, but otherwise felt to be performing at an average level at school. No seizures or unusual movements were reported at the time.

Over the next 4 years from age 7 to 11 years, visual acuity continued to decline to right 1.78 and left 2.3 logMAR (20/1200 and 20/4000). At this stage, the patient was relying on her extreme peripheral vision with difficulty maintaining steady fixation. Ultra-widefield fundus pseudocolor imaging demonstrated outer retinal atrophy from the vascular arcades to the far periphery, which had progressed since initial imaging (Figure 1, P1-D). Ultra-widefield–fundus autofluorescence showed corresponding hypo-AF nasally beyond the vascular arcades, and at the macula in a bull's-eye pattern (Figure 1, P1-E).

In October 2023 at age 11 years 8 months, miglustat was commenced and titrated up to 600 mg daily in 3 divided doses over 1 month. Her diet was modified to minimize disaccharides to avoid gastrointestinal side effects, and weight increased along previous centile. Side effects included only a minor tremor.

**Figure 2.**
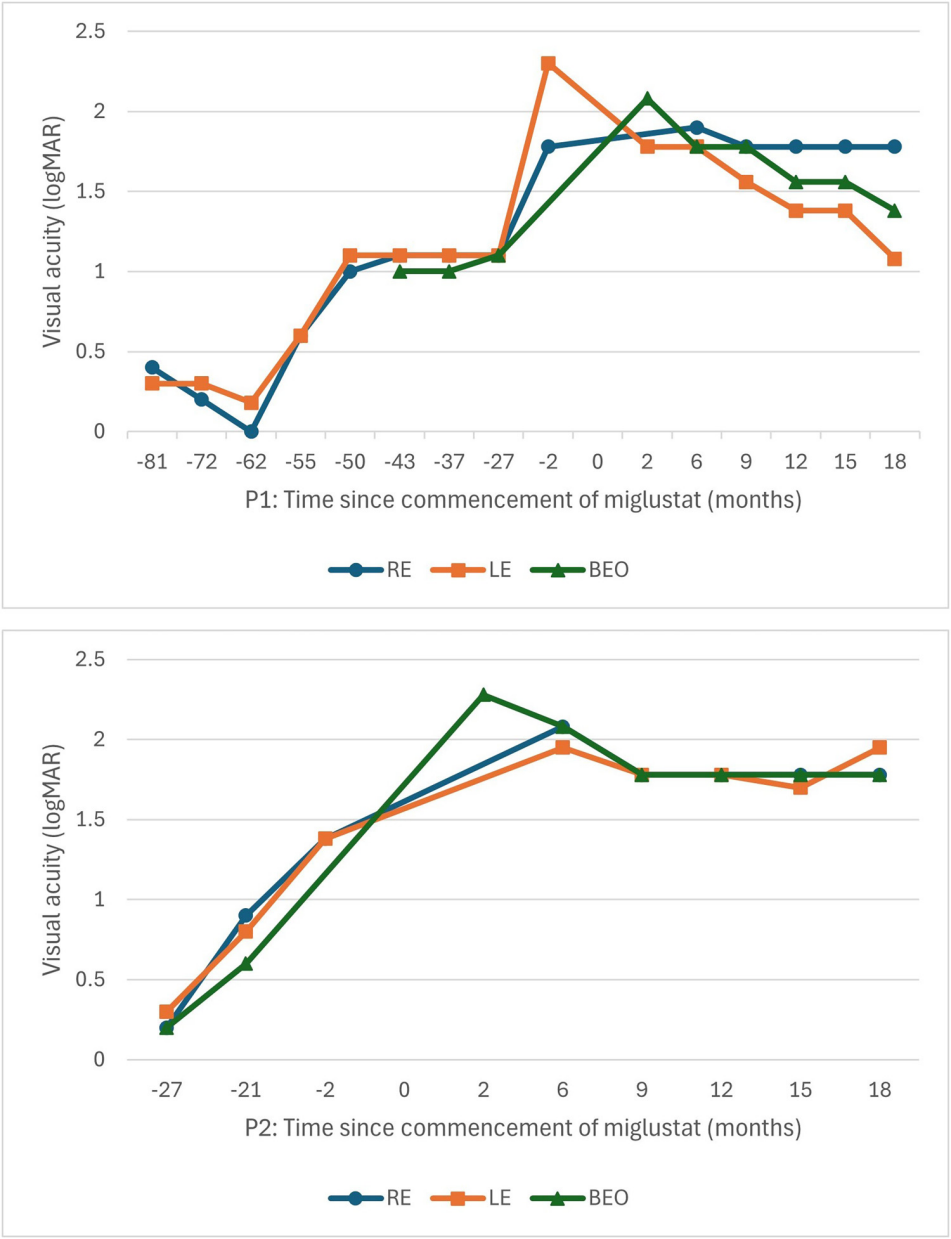
Visual Acuity Over Time, in Relation to Commencement of Miglustat for Patient 1 (P1) and Patient 2 (P2). RE, Right Eye; LE, Left Eye; BEO, Both Eyes Open.

At the 18-month review in April 2025, visual acuity measured right 1.78 and left 1.08 logMAR (20/1200 and 20/240), and 1.38 logMAR (20/480) with both eyes open (BEO), using peripheral fixation. Figure 2, P1, demonstrates visual acuity over time, in relation to commencement of miglustat. Clinical assessment noted a significant improvement in the patient's ability to hold a steady gaze. Optical coherence tomography imaging showed persistent maculae outer retinal disruption without significant change over the follow-up period, although the quality of the scans is limited by low central visual acuity and poor fixation (Figure 1, P1-F and P1-I). Ultra-widefield fundus pseudocolor imaging demonstrates stable appearance, but ultra-widefield–fundus autofluorescence highlights a gradual increase in hypo-FAF in the midperiphery extending inward to the vascular arcades compared with the imaging performed prior to starting miglustat (Figure 1, P1-D, P1-E, P1-G and P1-H).

**Figure 3.**
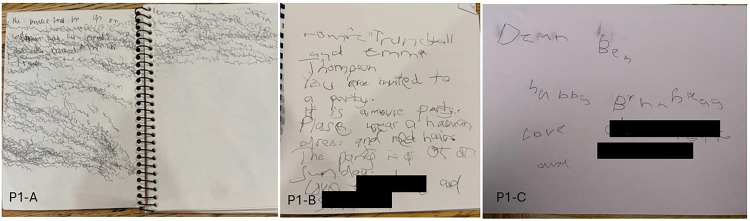
Handwriting of Patient 1 Prior to Miglustat (P1-A) and at 12 Months (P1-B) and 18 Months (P1-C) After Commencing Miglustat. Names are Deidentified.

Her parents reported improved visual behavior at home, including seeing and identifying her reflection in a window, identifying friends, and describing others’ facial features in detail. She regained her ability to identify objects and colors, type, and interact with visually complex games on a tablet computer and reach for objects in the inferior visual field. Her handwriting was more legible by 12 months following commencement of treatment, and similarly neat and controlled at 18 months (Figure 3). Her school performance has also improved, with parents and educators reporting notable improvements in her ability to retain and recall information. At 18 months since treatment commencement, she remains seizure free.

Vineland-3 Comprehensive Parent/Caregiver interviews were performed prior to use of miglustat and at 11 months and 18 months after treatment commencement. Scores were noted to have improved from baseline across all domains (Table 1). Changes were statistically reliable in 3 scores using the Jacobsen Reliable Change Index method.^16^ Unified Batten Disease Rating Scale scores were stable at 6-, 11-, and 17-month assessments with a trend for improvement in the Physical subscale, which decreased by 5 points from a score of 10 at baseline to 5 points at 17 months (Table 2).

**Table 1.** Vineland Adaptive Behavior Scales (Vineland-3) Assessment Over 18 Months of Treatment.^a^

	October 2023 Pretreatment			September 2024 (11-mo treatment)			April 2025 (18-mo treatment)			
	Score (95% CI)	PR (adaptive level)		Score (95% CI)	PR (adaptive level)		Score (95% CI)	PR (adaptive level)		*P* value (significance of reliable change)
Patient 1										
Adaptive Behavior Composite	74 (75-85)	4 (moderately low)		95 (92-98)	37 (adequate)		88 (85-91)	21 (adequate)		0.0116* (pre, 12 mo), 0.0924 (pre, 18 mo)
Communication	80 (75-85)	9 (moderately low)		102 (97-107)	55 (adequate)		91 (86-96)	27 (adequate)		0.0018** (pre, 12 mo), 0.1183 (pre, 18 mo)
Daily Living Skills	65 (60-70)	1 (low)		87 (82-92)	19 (adequate)		84 (79-89)	14 (moderately low)		0.0118* (pre, 12 mo), 0.0297* (pre, 18 mo)
Socialization	82 (78-86)	12 (moderately low)		100 (96-104)	50 (adequate)		98 (94-102)	45 (adequate)		0.0600 (pre, 12 mo), 0.0924 (pre, 18 mo)
Patient 2										
Adaptive Behavior Composite	101 (98-104)	53 (adequate)		105 (102-108)	63 (adequate)		102 (99-105)	55 (adequate)		0.6307 (pre, 12 mo), 0.9043 (pre, 18 mo)
Communication	107 (102-112)	68 (adequate)		113 (108-118)	81 (adequate)		107 (102-112)	68 (adequate)		0.3942 (pre, 12 mo), 1.0000 (pre, 18 mo)
Daily Living Skills	83 (78-88)	13 (moderately low)		95 (90-100)	37 (adequate)		95 (90-100)	37 (adequate)		0.1698 (pre, 12 mo), 0.1698 (pre, 18 mo)
Socialization	112 (108-116)	79 (adequate)		107 (103-111)	68 (adequate)		105 (101-109)	63 (adequate)		0.6014 (pre, 12 mo), 0.4645 (pre, 18 mo)

Abbreviations: CI, confidence interval; PR, percentile rank.

a Scores per Vineland-3 Comprehensive Parent/Caregiver Interview. Standard scores with 95% CIs, PRs, and adaptive level classifications are shown for each domain and the Adaptive Behavior Composite at baseline (October 2023), after 11 months (September 2024), and after 18 months (April 2025) of treatment. *P* values indicate the statistical significance of reliable change from baseline to 12 and 18 months.

*Significant at *P* <.05.

**Significant at *P* <.01.

**Table 2.** Unified Batten Disease Rating Scale (UBDRS) Assessments Over 17 Months of Treatment.

	Oct 2023; pretreatment		May 2024; 6 mo of treatment		Sept 2024; 11 mo of treatment		April 2025; 17 mo of treatment	
	Patient 1	Patient 2	Patient 1	Patient 2	Patient 1	Patient 2	Patient 1	Patient 2
Physical assessment	10	4	9	3	6	3	5	2
Behavior assessment	8	5	9	7	8	4	6	4
Capability: Normal vision	13	14	11	14	13	14	10	14
Capability: Actual vision	9	10	9	11	10	13	10	12
Global impression of change	-	-	3	2	2	2	3	3
CGI cognitive	2	1	2	1	2	1	2	1
CGI behavior	1	1	2	2	1	1	1	1
CGI mood	1	2	2	2	1	1	1	1
CGI motor	2	1	1	1	1	1	1	1
CGI overall	2	2	3	2	2	2	2	2

Scores are presented across domains of physical function, behavior, visual capability, and clinician-rated global impressions at baseline (October 2023) and at 6, 11, and 17 months post treatment initiation. Higher scores reflect greater disease severity. Clinical Global Impression (CGI) subscales include cognition, behavior, mood, motor function, and overall severity.

### Patient 2

The younger sister of patient 1 at age 7 years 0 months was noted to have altered visual behavior manifesting by holding a tablet computer closer to her face and some difficulty reading the whiteboard at school.

At presentation, visual acuity was right 0.2 and left 0.3 logMAR (20/30 and 20/40). Ocular motility was normal and there was no strabismus. Her cycloplegic refraction was right +0.50 and left +0.50. Bull's-eye maculopathy was confirmed on funduscopy and optical coherence tomography imaging (Figure 1, P2-A).

She was referred for targeted gene analysis which confirmed homozygosity for the *CLN3* common 1 kb deletion (c.461-280_677+382del966), consistent with a diagnosis of CLN3 disease. Systemically, anxiety and mild behavioral changes had been noted. There had not been any seizures or unusual movements.

By August 2023, at the age of 9 years 1 month, visual acuity had reduced to 1.38 logMAR (20/480) right and left eyes. Ultra-widefield fundus pseudocolor imaging and ultra-widefield–fundus autofluorescence revealed significant retinal atrophy in the posterior poles in both eyes (Figure 1, P2-B and P2-C). Optical coherence tomography imaging of the maculae confirmed outer retinal atrophy and significant disruption of the EZ (Figure 1, P2-D).

Miglustat was commenced in October 2023 at age 9 years 3 months, and increased up to 400 mg daily in 3 divided doses over 1 month.^10^ She has not experienced side effects, with similar dietary modification, and her weight has increased along the same centile.

At the 3-month review, visual acuity was recorded as “hand motion” with BEO and converted to 2.28 logMAR (20/3800).^13^ At 18 months, visual acuity was measured at right 1.78 and left 1.95 logMAR (20/1200 and 20/1780), and 1.78 logMAR (20/1200) BEO using peripheral fixation. Figure 2, P2, charts visual acuity over time, in relation to commencement of miglustat. Similar to her sister, she was now able to hold her eyes steady most of the time but relied on her inferior visual field for visual tasks. Ultra-widefield pseudocolor imaging and optical coherence tomography maculae demonstrated stable appearances over the past 18 months (Figure 1, P2-B, P2-E, P2-D, and P2-G), although there was an increase in the area of hypo-AF on ultra-widefield–fundus autofluorescence in the midperiphery extending to the macula (Figure 1, P2-C and P2-F). She was able to distinguish color, whereas loss of color perception had been reported approximately 18 months earlier. By the end of the assessment period, she remained relatively photophobic and regularly wore sunglasses.

Her parents reported a significant improvement in visual and motor functioning since commencing miglustat, including being able to ride a bike again, scootering, skipping along the footpath, and engaging in indoor rock-climbing. Her academic performance had also improved, with parents and educators noting better information retention and recall, increased fluency in her reading and writing, and overall improvement in academic performance commensurate with classroom peers.

Her Vineland-3 (Table 1) and Unified Batten Disease Rating Scale (Table 2) scores were stable with a trend for improvement in daily living skills that did not meet significance for change. Similarly, the Unified Batten Disease Rating Scale scoring pertaining to actual vision capability appeared to have improved. At 18 months since treatment commencement, she remained seizure free.

There were no changes in allied health therapies for either patient when comparing the period before starting miglustat to the current period. Patient 1 continued to receive speech and language therapy, and both patients received classroom-based vision support. Neither patient was taking any concomitant medications.

## Discussion

Neuronal ceroid lipofuscinosis (NCL), or Batten disease, is a heterogenous group of conditions that are collectively the most common neurodegenerative condition in children.^17^ CLN3 disease is the most prevalent of the 13 subtypes, accounting for more than 30% of all NCL cases. Depending on geographical region, the incidence of CLN3 disease ranges from 1 in 100 000 live births to 1 in 21 000, with the highest prevalence in Scandinavian countries.^18–20^

CLN3 disease is caused by variations in the *CLN3* gene encoding for a transmembrane protein of 438 amino acids localized in endosomal/lysosomal compartments, plasma membranes, and in synaptosomes.^21^ In 70% to 85% of patients, the pathologic variant is a homozygous 1-kb deletion of 2 exons 7 to 8 on chromosome 16p11.2., predicted to result in a truncated protein,^21,22^ although the functional impact of this and other rarer variants on CLN3 transcription and disease pathogenesis remains elusive.^23^ Although the function of the CLN3 protein is not yet fully understood, emerging evidence suggests it plays a critical role in lipid homeostasis, particularly in the lysosomal clearance of glycosphingolipids including globotriaosylceramide (Gb3)^24,25^ and glycerophosphodiesters.^26,27^ Specifically, accumulation of Gb3 has been observed in patient postmortem tissue and CLN3-deficient human and mouse cell lines,^26–28^ and significantly increased levels of ganglioside GM3 have been reported in mouse cell lines.^28,29^ Elevated glycerophosphodiester levels have been detected in various CLN3-deficient models, including yeast cells, mouse tissue, serum from both mice and pigs, cultured human cells, human cerebrospinal fluid, and plasma.^26,27,30^ Studies in patient-derived CLN3 stem cell lines and in vivo also suggest that the CLN3 protein is directly linked to phagocytic function in RPE cells, which is critical to managing the renewal of the photoreceptor outer segment discs and hence visual function.^31,32^ Furthermore, decreased RPE autofluorescence/lipofuscin is shown to coincide with the earliest time point of scotopic and photopic visual deficit and precedes the loss of photoreceptor outer segment and photoreceptors in these preclinical models.^32^

Recently, miglustat has been investigated as a potential substrate reduction therapy intervention for CLN3 disease. Miglustat is a small molecule inhibitor of glucosylceramide synthase that is able to cross the blood-brain barrier and inhibit the downstream synthesis of glycosphingolipids.^28^ In cell and animal models of CLN3 disease, miglustat treatment is associated with reduced accumulation of glycosphingolipid and mitochondrial ATP synthase subunit c, reduced excitotoxicity, and normalized cellular function.^25^

A phase I/II trial of miglustat for the treatment of CLN3 disease was recently completed in 6 patients aged 17 years and older.^11,12,33^ Primarily designed to investigate safety and tolerability, some efficacy measures were included in this study, including neurologic (Unified Batten Disease Rating Scale) and biomarker assessments in plasma and cerebrospinal fluid. Given complete vision loss is typically observed from around 10 to 11 years of age,^2,3,5^ ophthalmologic assessments were listed as a secondary outcome.

Preliminary 12-month results demonstrated an average 32% decline from baseline in serum neurofilament light chain, 64% reduction in cerebrospinal fluid neurofilament light chain, and 45% reduction in serum glycosphingolipids, specifically globotriaosylceramide (Gb3). Clinically, motor function remained stable over 12 months of treatment, with no notable change in the modified Unified Batten Disease Rating Scale physical assessment subscale score compared with age-matched natural history controls.^11^ After 18 months of treatment, motor stabilization continued, with a mean change in Unified Batten Disease Rating Scale physical assessment core of +1.83 compared to +6.04 in age-matched natural history controls (n = 46) (where higher score denotes greater motor progression), together with a continued favorable safety and tolerability profile.^34^

In 80% of individuals with syndromic CLN3 disease, vision impairment is the first presenting symptom.^8^ Multiple independent natural history studies across discrete international patient cohorts report first onset of vision loss at around age 5.4 ± 1.4 years (mean ± standard deviation) to 6.5 ± 1.1 years^2–7^ with complete vision loss around 10.7 ± 4 years to 12.4 ± 2 years.^2–7^ These data suggest little variance in the visual phenotype in syndromic CLN3 disease population and are consistent with the age of onset of vision loss observed in both patients reported here before commencement of miglustat treatment.

The CLN3 disease ocular phenotype has been characterized in 2 independent patient cohorts with genetically confirmed CLN3 disease.^35,36^ Retinal degeneration is typically observed to progress in a centrifugal pattern, starting with central hypo-AF and a surrounding ring of hyper-AF on ultra-widefield–fundus autofluorescence. As the disease progresses, the whole macula region shows generalized hypo-AF.^36^ Similarly, optical coherence tomography imaging demonstrates progression of disease from early foveal EZ disruption to eventually significant EZ disruption and difficulty identifying any remaining outer retinal structures.^36^

It is relevant to note that in practice, assessment of visual acuity can be challenging in this patient cohort because of poor visual function and increasing behavioral and cognitive changes. Obtaining a thorough history of visual behavior during activities of daily living is an invaluable component in the overall understanding of a patient's response to treatment.

Here, the outcome measures of response to miglustat treatment included comprehensive ophthalmologic assessment, Unified Batten Disease Rating Scale and Vineland-3 assessments.

Our experience of miglustat use in 2 young patients with CLN3 disease demonstrates promising results. Ophthalmologically, there has been an improvement in visual behavior but with likely gradual disease progression on multimodal imaging, suggesting a structure-function mismatch. Given the improvement in visually guided tasks described in these cases, it is tempting to postulate that the initial results seen here in these younger patients may be due to the timing of treatment at a relatively early stage of the disease course where, despite visual decline, the retina remains somewhat structurally and mechanistically intact and amenable to rescue of pathologic glycerophosphodiester and glycosphingolipid storage. It is possible, however, that improvements in visual function may, in part, be a result of enhanced cognitive function and ability to undertake the test. In other inherited metabolic diseases with retinal involvement where systemic disease-modifying therapies exist—such as dietary interventions or pharmacologic treatment^37^—neurologic symptoms may stabilize or improve, whereas retinal degeneration may be less responsive. For example, in cobalamin C deficiency, dietary modification can lead to systemic improvement, but retinal function frequently continues to decline. This may reflect, in part, the fact that once retinal degeneration has occurred, restoration is not possible^38^ Longer-term follow-up of the two patients presented here is essential to evaluate the durability of these early visual gains and to better understand the potential therapeutic impact of miglustat on the respective retinal and neurologic manifestations of CLN3 disease.

The improvements on Vineland-3 testing reflect the family's described experience of the girls’ improvement on a day-to-day basis. These improvements, along with other functional and cognitive gains reported by educators, such as a notable increase in handwriting legibility (Figure 3), are contrary to the well-documented trajectory of cognitive decline reported in large natural history studies,^5,6^ including a typically precipitous loss of writing ability around age 11-13 years.^5^

Unified Batten Disease Rating Scale scores (where higher scores indicate greater disease severity) remained stable across 6-, 11-, and 17-month assessments for both patients. Patient 1 showed a 5-point decrease (improvement) in the physical subscale over this period. In contrast, longitudinal studies of CLN3 cohorts report a near-linear annual increase in Unified Batten Disease Rating Scale physical subscale scores, with mean rates of +2.86 points/year (95% confidence interval: 2.27-3.45; n = 82)^35^ and +3.11 ± 0.28 points/year (n = 79).^36^

Although these findings are encouraging, we acknowledge several important limitations. The open-label design, small sample size (n = 2), and relatively short follow-up period limit the robustness and generalizability of the results. Subjective reports of benefit from caregivers may be subject to bias, particularly given the family's considerable effort and financial burden in accessing miglustat. However, these caregiver observations align with improvements seen in objective ophthalmologic and neurologic assessments, lending additional credibility to the findings.

Although the 18-month follow-up offers preliminary insight into the disease-modifying effect of miglustat therapy in a pediatric cohort, it remains unclear whether its impact is sustained or whether disease progression will resume over time. The rarity of CLN3 disease further complicates efforts to conduct adequately powered studies. Nonetheless, when objective improvements are observed in the context of a condition with high unmet need and no established treatment, it is critical to report such cases. These early findings will also serve to guide hypothesis generation and inform the design of future research.

Larger, prospective studies with extended follow-up, blinded outcome assessment, and either placebo control or comparison with age-matched natural history cohorts will be necessary to more definitively assess the efficacy of miglustat in this population. These results highlight the importance of early diagnosis of CLN3 disease that can help facilitate earlier treatment when maximal benefit and patient outcomes may be obtained.^35,36^ As we await further developments in the clinical trial of miglustat treatment for CLN3 disease, these two patients continue to undergo frequent ophthalmic and neurologic assessments to monitor disease progression.

## Conclusion

This case series describes notable improvements in visual function and stabilization of neurologic decline in 2 pediatric patients with CLN3 disease treated with miglustat over an 18-month period. To our knowledge, this is the first report of clinical outcomes following miglustat treatment in pediatric patients (under 17 years of age), which may represent a disease stage potentially more responsive to intervention.

Interestingly, a structure-function mismatch was observed, with measurable improvements in visual function occurring despite gradual disease progression seen in retinal structure on multimodal imaging. This highlights the complexity of monitoring treatment response in CLN3 disease. Given the progressive psychosocial and neurologic impairment associated with the disorder, formal vision testing can be challenging; however, detailed caregiver-reported histories of functional vision and activities of daily living may provide valuable context and insight into clinically meaningful changes.

These early findings support the need for further investigation through controlled clinical trials in pediatric populations, with extended follow-up to assess long-term efficacy and durability of response to miglustat in CLN3 disease.
